# Supplementary material for: Liensinine Prevents Acute Myocardial Ischemic Injury via Inhibiting the Inflammation Response Mediated by the Wnt/β-Catenin Signaling Pathway
Source: Int J Mol Sci. 2025 May 10;26(10):4566. doi: 10.3390/ijms26104566 (PMC12110967; doi:10.3390/ijms26104566)
Supplement: Supplementary file 1 [file ijms-26-04566-s001.zip › ijms-3570261-supplementary.pdf]

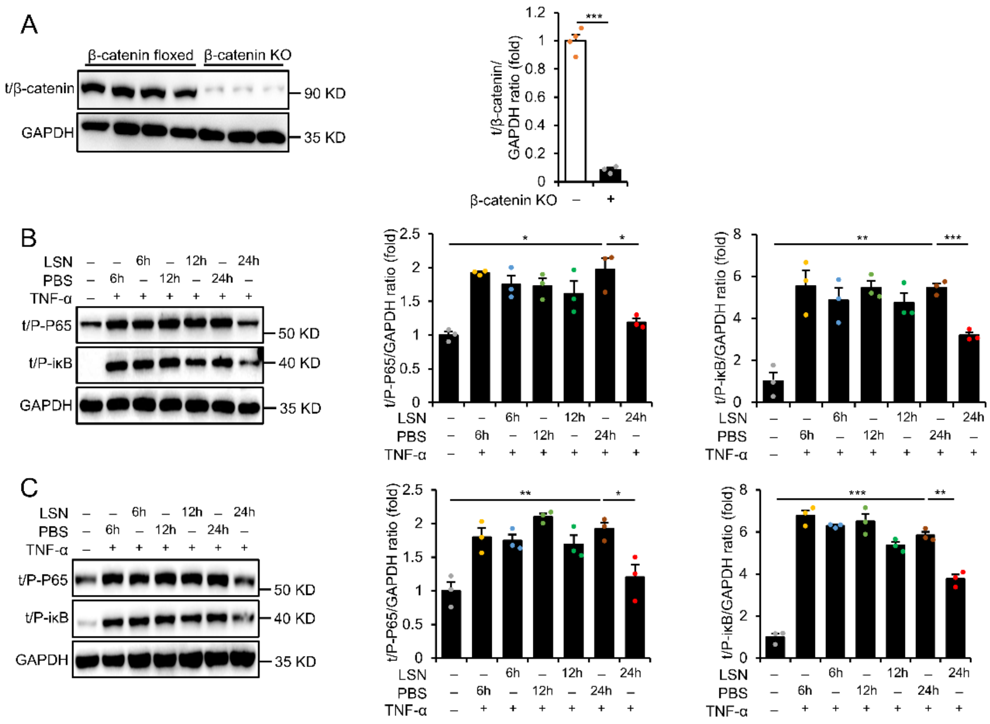

Figure S1. LSN prevents TNF-α-induced inflammatory responses

(A) Representative immunoblots (left) and quantification (right) of total β-catenin in the heart at 4 weeks following tamoxifen administration (30 mg/kg/day for 5 days) in β-cateninfl/fl and α-MHC-Cre-β-catenin KO mice. n=3 or more. (B) Representative immunoblots (left) and quantification (right) of total P-P65 and P-iκB expression in H9C2 cardiomyocytes induced by TNF-α treatment for 12 h following pretreatment with LSN for 6 h, 12 h or 24 h. n=3. (C) Representative immunoblots (left) and quantification (right) of total P-P65 and P-iκB expression in AC16 cardiomyocytes induced by TNF-α treatment for 12 h following pretreatment with LSN for 6 h, 12 h or 24 h. n=3. Data are presented as mean ± SEM; * $p < 0.05$, ** $p < 0.01$, *** $p < 0.001$.

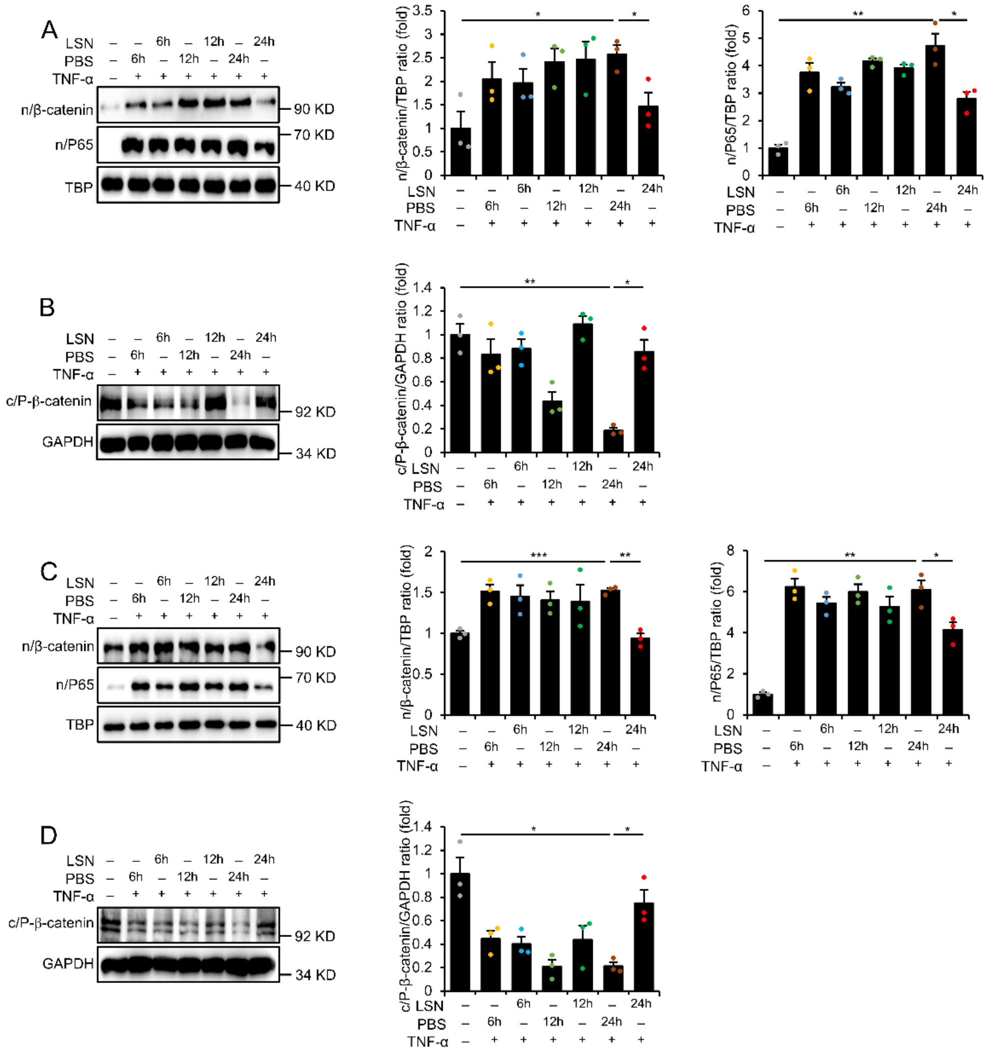

Figure S2. LSN prevents TNF-α-induced Wnt/β-catenin activation.

(A) Representative immunoblots (left) and quantification (right) of nuclear β-catenin, P65 expression in H9C2 cardiomyocytes induced by TNF-α treatment for 12 h following pretreatment with LSN for 6 h, 12 h or 24 h. n=3. (B) Representative immunoblots (left) and quantification (right) of cytoplasmic phospho-β-catenin expression in H9C2 cardiomyocytes induced by TNF-α treatment for 12 h following pretreatment with LSN for 6 h, 12 h or 24 h. n=3. (C) Representative immunoblots (left) and quantification (right) of nuclear β-catenin, P65 expression in AC16 cardiomyocytes induced by TNF-α treatment for 12 h following pretreatment with LSN for 6 h, 12 h or 24 h. n=3. (D) Representative immunoblots (left) and quantification (right) of cytoplasmic phospho-β-catenin expression in AC16 cardiomyocytes induced by TNF-α treatment for 12 h following pretreatment with LSN for 6 h, 12 h or 24 h. n=3.Data are presented as mean±SEM, *P<0.05, **P<0.01, ***P<0.001.

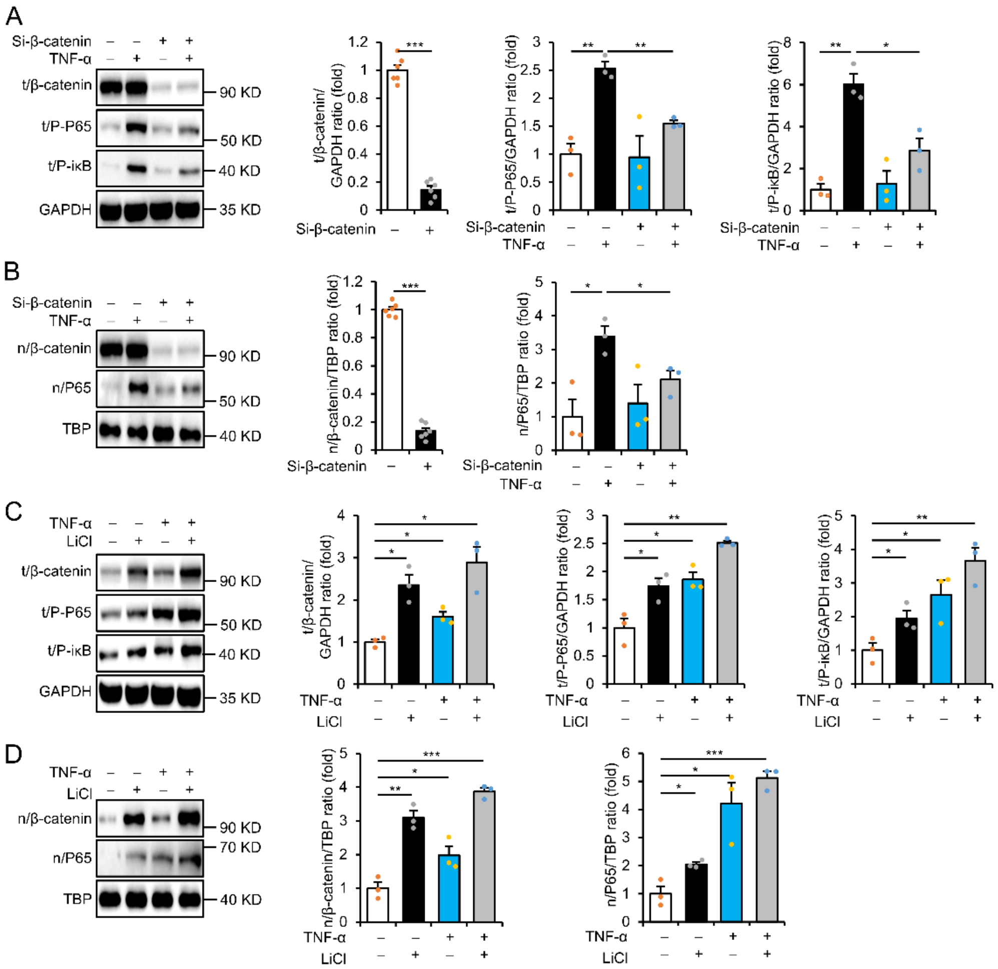

Figure S3. Knockdown or activation of β-catenin in TNF-α-induced inflammatory responses. (A) Representative immunoblots (left) and quantification (right) of total β-catenin, P-P65 and P-iκB expression following siRNA-mediated knockdown of β-catenin for 48 h, subsequent treatment with TNF-α for 12 h in AC16 cardiomyocytes. n=3. (B) Representative immunoblots (left) and quantification (right) of nuclear β-catenin and P65 expression following siRNA-mediated knockdown of β-catenin for 48 h, subsequent treatment with TNF-α for 12 h in AC16 cardiomyocytes. n=3. (C) Representative immunoblots (left) and quantification (right) of total β-catenin, P-P65 and P-iκB expression following TNF-α or LiCl treatment for 12 h in AC16 cardiomyocytes. n=3. (D) Representative immunoblots (left) and quantification (right) of nuclear β-catenin and P65 expression following TNF-α or LiCl treatment for 12 h in AC16 cardiomyocytes. n=3. Data are presented as mean±SEM, *P<0.05, **P<0.01, ***P<0.001.
